# Visuomotor Control Accuracy of Circular Tracking Movement According to Visual Information in Virtual Space

**DOI:** 10.3390/s25195998

**Published:** 2025-09-29

**Authors:** Jihyoung Lee, Kwangyong Han, Woong Choi, Jaehyo Kim

**Affiliations:** 1 Department of Industrial & Management Engineering, Pohang University of Science and Technology, Pohang 37673, Republic of Korea; iwoneye@postech.ac.kr; 2 Department of Mechanical and Control Engineering, Handong Global University, Pohang 37554, Republic of Korea; 22474002@handong.ac.kr; 3 Division of ICT Convergence Engineering, Kangnam University, Yongin 16979, Republic of Korea

**Keywords:** circular tracking movement, circular tracking movement evaluation system, control accuracy, multisensory integration, virtual reality

## Abstract

The VR-based circular tracking movement evaluation system (CES) was developed to quantitatively assess visuomotor control. The virtual stick, a component of the CES, provides visual cues in the virtual environment and haptic feedback when holding the controller. This study examined the effects of stick presence and presentation order on control accuracy for distance, angle, and angular velocity. Twenty-seven participants (12 females; mean age 23.3 ± 2.3 years) performed tasks in the frontal plane followed by the sagittal plane. In each plane, the stick was visible for the first 1–3 revolutions and invisible for the subsequent 4–6 revolutions in the invisible condition, with the reverse order in the visible condition. In the invisible condition, control accuracy with the stick was 1.10 times higher for distance only in the sagittal plane, and 1.13 and 1.09 times higher for angle and angular velocity in the frontal plane, and 1.11 and 1.08 times higher in the sagittal plane. No significant differences were observed in the visible condition. The improved control accuracy when the stick was visible is likely due to enhanced precision in constructing the reference frame, internal models, body coordinates, and effective multisensory integration of visual and haptic information. Such visual information may enable fine control in virtual environment-based applications, including games and surgical simulations.

## 1. Introduction

Visuomotor control plays a crucial role in learning through the imitation of visually guided actions in human tasks and daily activities. We naturally learn by observing and mimicking the movements of others in workplace or residential space, such as using tools, choreographed movements, and sports techniques. In the brain, however, complex computations of visual information and the consequent motor control are simultaneously executed [1,2,3,4,5]. Humans are known to utilize predictive-control and feedback-control mechanisms based on body coordinates to control their limbs [6,7,8,9]. Human control mechanisms are implemented through the sensorimotor integration system [10], where the reference body coordinates are established by an internal model that tracks the changes between the target position and the current position, and by collecting proprioceptive sensory information from muscles, tendons, and joints [11,12]. In neuroscience, it has been proposed that reference frames for specifying the positions of the body and objects and for planning movement are supported by grid cells and by place cells that process multimodal sensory information [13,14,15,16,17,18,19,20]. Both cell types are considered necessary for perceiving objects and the surrounding environment and for generating the body coordinate system that serves as the reference within the sensorimotor integration system.

Research on visuomotor control has implemented visual target trajectories of different dimensionalities to characterize upper-limb movement. One-dimensional (1D) target trajectories have typically been realized as straight or sinusoidal paths directed toward a goal [21,22,23,24], whereas two-dimensional (2D) trajectories have been configured as circular paths in the frontal plane and used for tracking analyses [25,26,27,28,29]. Because human movement inherently occurs in three-dimensional space, subsequent studies extended tracking analyses to target trajectories that included the sagittal plane [30]. Circular tracking movement (CTM) not only shares the periodicity of a 1D sinusoid but also, through continuous movement at approximately constant speed, enables the analysis of visuomotor control characteristics in two and three dimensions [31,32]. CTM permits precise analysis using polar kinematic parameters and allows evaluation of control accuracy for distance (R), angle (θ), and angular velocity (ω) relative to a reference [10,31,32,33,34,35]. A comprehensive understanding of visuomotor control requires parameter analyses across diverse conditions such as speed, acceleration, monocular versus binocular viewing, and dominant versus nondominant hand use. However, a quantitative evaluation system to support such analyses has been lacking.

Choi et al. (2018) [31] developed a CES designed to quantitatively analyze three-dimensional visuomotor control characteristics based on CTM within a VR environment. The CES comprises basic and variable elements, allowing for the configuration of experimental conditions in the VR environment. The basic elements include four components: circle (visible/invisible), target, tracer, and stick. The variable elements include the plane of the body, speed, direction, acceleration, among other factors that can be adjusted or added. Notably, the stick, as one of the basic elements, acts as a mediator that provides visual information in the VR environment and simultaneously connects the tactile feedback from holding the VR device’s controller in the real world. By configuring the states or conditions of both the basic and variable elements in various combinations, CES enables the study of visuomotor control characteristics from multiple perspectives.

Previous studies using the CES have evaluated control accuracy, control strategies, and transient responses under various visuomotor conditions, including monocular or binocular vision, body orientation, target speed, hand dominance, axis of rotation, and the presence or absence of depth information (Table 1) [10,31,32,33,34,35,36]. These studies have contributed to understanding motor control mechanisms in CTMs. However, to extend the CES to precise control characteristic analysis, a thorough assessment of control accuracy for all components is necessary. One of the basic elements, the virtual stick is a connecting mediator that provides visual information in the VR environment and tactile information from holding the VR device’s controller in the real world. The stick is designed as a 20 cm long rod with its angle synchronized to that of the HMD controller. Participants are expected to perceive the stick as having a tracer attached to its end, similar to the real-world controller. Additionally, participants are anticipated to consider themselves as holding the virtual stick in the VR environment, equating the stick with their own hand. Without the stick, it would be challenging for participants to determine their hand’s position in the VR environment or to discern whether the hand holding the controller is positioned in front, on the side, or above the tracer, which could lead to increased discrepancies in the distance, angle, and angular velocity of the tracer relative to the target.

The first hypothesis posited that the presence of the 20 cm stick within CES would increase control accuracy. Although tactile information is available through the hand holding the controller, we reasoned that adding visual information would enable more precise motor control. The second hypothesis posited that the presentation order of the stick during CTM, in which the stick is initially present and then absent or initially absent and then present, would influence control accuracy. From a multisensory integration perspective, we expected control accuracy to decrease when part of the visual information is lost while visual and tactile cues are concurrently available, which would disrupt the integrated state, and to increase when visual information is added while tactile input is present, which would reconstitute multisensory integration.

Based on these assumptions, the present study employed CES to analyze differences in visuomotor control accuracy as a function of the presence of the stick, which serves as a mediator linking visual and tactile information. First, we compared conditions with and without the stick by analyzing differences in the polar kinematic parameters ΔR, Δθ, and Δω. Next, we examined differences in control accuracy and its stability across presentation order using the same parameters.

## 2. Materials and Methods

### 2.1. Research Framework

**Figure 1:**
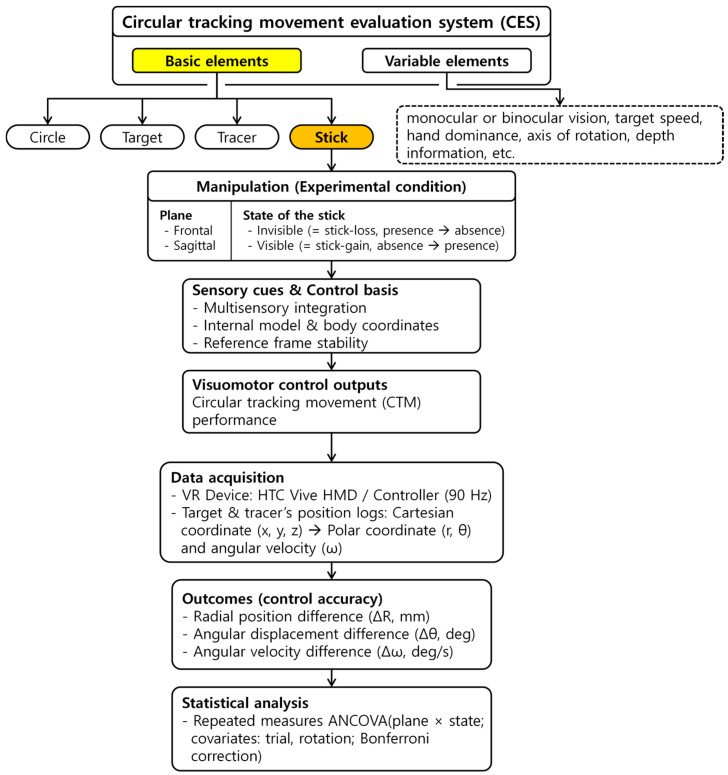
Research framework of the present study. The basic elements of the CES are indicated in yellow, and the stick, highlighted in orange, was used to define the experimental conditions according to body plane and the state of the stick (invisible/visible). Visuomotor control outputs in circular tracking movement (CTM) was quantified by computing differences in terms of radial (ΔR), angular (Δθ), and angular velocity (Δω) between the target and tracer, and control accuracy across conditions was compared using repeated-measures ANCOVA.

The present study was conducted following the framework shown in Figure 1. The basic elements of the CES allow for systematic control of visual availability. In this study, experimental conditions were established in which the stick was either invisible (stick-loss) or visible (stick-gain) in both the frontal and sagittal planes of the body. The visual presence or absence of the stick in the VR environment was expected to influence sensory signals and the basis of control, including multisensory integration, internal models, body coordinates, and reference frame stability, ultimately leading to changes in visuomotor control output as reflected in CTM performance. To examine these changes, Cartesian coordinates (x, y, z) of the target and tracker were collected through VR equipment and converted into polar coordinates for CTM characteristic analysis. Based on the target position, the differences between the target and tracer were calculated to derive radial (ΔR), angular (Δθ), and angular velocity (Δω) variables for evaluating control accuracy. These outcomes were then subjected to repeated-measures ANCOVA to compare control accuracy depending on the presence or absence of the stick.

### 2.2. Participants

This study involved 27 participants (female: 12) and their mean age was 23.3 ± 2.3 (*SD*) years and all of them were right-handed. All participants were selected based on normal visual acuity, determined to be 0.8 or higher, either unaided or corrected, according to health examination results. Participants who had previously participated in similar studies or exhibited cybersickness in virtual environments were excluded from the study. All participants signed a webpage or printed consent form before prior to the experiment. All aspects of the study, including minor details, were implemented following the research plan and in accordance with ethical standards approved by the Institutional Review Board (IRB) of Handong Global University (Approval No. 2023-HGURA020). The recruitment of participants was conducted from 4 October 2023, the date of IRB approval, to 31 December in the same year.

### 2.3. Experimental Task

The experiment was conducted using the CES, which was designed to allow the configuration of the stick’s visibility in the VR environment. The CTM evaluated in the CES was designed for participants to track a target (Ø 30 mm) moving along a circular trajectory of Ø 300 mm in the virtual space while wearing the HTC VIVE HMD, using a tracer (Ø 20 mm) controlled by the HTC VIVE controller. The stick was represented as a line connected to the center of the tracer at the same angle as the controller, and its length was set to 200 mm. The x, y, and z coordinates (mm) of the target and tracer in the CES were recorded at a sampling rate of 90 Hz using the HTC VIVE HMD.

**Figure 2:**
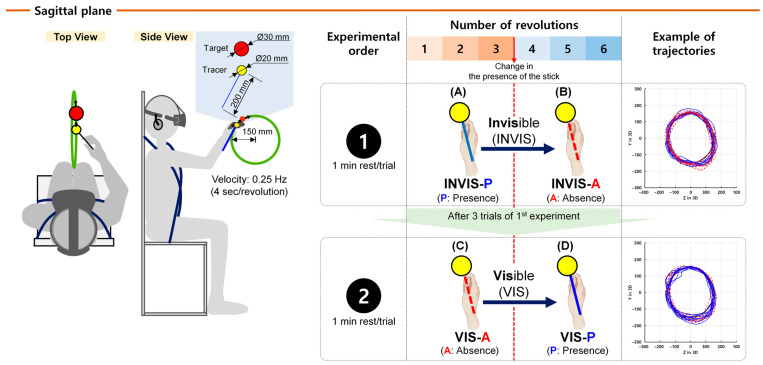
Experimental procedure. The experiment was conducted first in the frontal plane followed by the sagittal plane. The left side of the figure shows the sagittal plane setup. In each plane, participants performed the invisible (INVIS) condition first, followed by the visible (VIS) condition. Each condition consisted of three trials. A rest period of approximately 1 min was provided between trials. Each trial consisted of six revolutions, and the presence of the stick changed starting from the fourth revolution. After completing all three trials of the INVIS condition, participants proceeded to the VIS condition. Here, P refers to the presence of the stick and A refers to the absence of the stick. (**A**) In the INVIS condition, revolutions 1–3 where the stick was present are labeled INVIS-P. (**B**) Revolution 4–6 where the stick was absent are labeled INVIS-A. (**C**) In the VIS condition, revolution 1–3 where the stick was not visible are labeled VIS-A. (**D**) Revolutions 4–6 where the stick was visible are labeled VIS-P. The blue solid line represents the tracer’s trajectory under the presence (P) of the stick, whereas the red dashed line represents the tracer’s trajectory under its absence (A).

The experiment to compare control accuracy based on the presence and visual presentation order of the stick was conducted according to the procedure illustrated in Figure 2. The study was carried out on the frontal plane, where only position information varied, and on the sagittal plane, where both position and depth information changed. Participants performed a total of six revolutions of CTM. Two experimental conditions were defined. In the invisible condition (INVIS), the stick was present during the first three revolutions and was absent from the fourth revolution onward. In the visible condition (VIS), the stick was initially absent and present starting from the fourth revolution. Within each condition, the presence or absence of the stick was further classified as presence (P) or absence (A), respectively. By combining these designations, four sub-conditions were defined. In the INVIS condition, the stick was present during revolutions 1 to 3, which were classified as the INVIS-P sub-condition, and absent during revolutions 4 to 6, which were classified as INVIS-A. In the VIS condition, the stick was absent during revolutions 1 to 3, corresponding to the VIS-A sub-condition, and present during revolutions 4 to 6, corresponding to VIS-P.

Once the evaluation of CTM using CES commenced, a countdown was initiated for 3 s, after which participants were required to manipulate the controller to track the moving target with the tracer. The target moved at 0.25 Hz (i.e., 90° of revolution per second), completing one revolution every 4 s. Before the experiment, participants were given sufficient time to familiarize themselves with the HTC Vive HMD and controller and to adapt to the motion of the target and tracer in the VR environment. Participants performed the task first in the frontal plane and then in the sagittal plane. Within each plane, the INVIS condition was performed first, followed by the VIS condition, and each condition comprised three repetitions before proceeding to the next. Approximately 1 min of rest was provided between trials. Because the CES task was designed to assess upper-limb movements such as shoulder and arm motion, it was conducted in a dedicated chair equipped with fixation belts that immobilized the torso, as illustrated on the left in Figure 2, to minimize the influence of movements of other body segments.

### 2.4. Data Analysis

For the purpose of data analysis, Cartesian coordinate data (x, y, z) for the target and tracer were transformed into polar coordinates, specifically the radial position “R” and angular displacement “θ”. Furthermore, angular velocity “ω”, denoting the rate of change in “θ” over the sample rate period, was also analyzed.

Radial position difference (ΔR), angular displacement difference (Δθ), and angular velocity difference (Δω) are widely utilized parameters in the analysis of CTM, as represented by the absolute difference in positions of the tracer relative to the target, as shown in Table 2 [10,31,32,33,34,35,36]. ΔR represents the difference in radius length (mm) from the origin to the target and from the origin to the tracer (Table 2). ΔR indicates the performance of maintaining the circular trajectory during the circular tracking movement and serves as a measure of spatial motion control characteristics [34]. Δθ signifies the absolute difference in angles (deg, °) between the target and the tracer with respect to the y-axis. Meanwhile, Δω indicates the absolute difference in angular velocity (deg/s) between the target and the tracer over a unit time (i.e., the sampling rate), reflecting the rate of change in angles. Δθ and Δω are parameters that reflect the angular deviation of the tracer in relation to the target during CTM, representing control performance in terms of position and velocity, respectively (Table 2), and they serve as indicators of temporal motion control characteristics [34]. In the calculation of ΔR, Δθ, and Δω, the x and y coordinate values were used for the frontal plane, while the y and z coordinate values were utilized for the sagittal plane.

To analyze differences in ΔR, Δθ, and Δω according to the presence or absence of the tracer stick during CTM performance, a two-way repeated-measures ANCOVA was conducted. The independent variables were plane (frontal vs. sagittal) and state (stick presence vs. absence), trial and revolution were included as covariates to control for any potential confounding effects. The effect of trial was not observed for any parameter, whereas the effect of revolution was statistically significant for Δθ and Δω. To determine the analytical range, ΔR, Δθ, and Δω were examined for each revolution. The results indicated that Δθ and Δω in the first revolution contained an initial high error rate, which is commonly observed during early performance [37]. Interpreting the results without adjustment would lead to an overestimation of control accuracy improvements in subsequent revolutions compared to the first revolution; therefore, the first revolution was excluded from analysis. Specifically, in INVIS-P and VIS-A, where the first revolution was included, only the data from the second and third revolutions were analyzed. In the corresponding sub-conditions INVIS-A and VIS-P, the fourth revolution within revolutions 4 to 6 was excluded because it coincided with the transition in the visual state of the stick. To ensure comparison under more stable conditions, only revolutions 5 to 6 were included in the analysis. This selection was justified by preliminary confirmation that the results from revolutions 5 to 6 in INVIS-A and VIS-P did not differ significantly from those of revolutions 4 to 6 or 4 to 5 (Table S5). For example, in the INVIS condition, the first revolution (R1) within INVIS-P and the first revolution (R4) within INVIS-A were excluded from the analysis. Consequently, of the six revolutions in each condition, the first and fourth revolutions were excluded from analysis. Main effects and interaction effects of the plane and state factors on each parameter (ΔR, Δθ, Δω) were statistically examined using SPSS Statistics V21 (IBM Corp., Chicago, IL, USA). Post hoc pairwise comparisons were performed using Bonferroni-corrected tests. From the analysis, mean values (M), standard errors (SE), and standard deviations (SD) were calculated. To validate the assumptions of repeated-measures ANCOVA, Mauchly’s test of sphericity was conducted. Results from post hoc tests were considered statistically significant at *p* < 0.05, and highly significant at *p* < 0.01. The outcomes of the sphericity test, as well as the effect sizes for plane and state factors, are reported in Tables S1, S2 and S3 using partial *η*^2^ and Cohen’s *d*, respectively.

We conducted an a priori power analysis using G*Power software version 3.1.9.7 to determine the minimum sample size required for repeated-measures and within-between interaction ANCOVA [38]. Using the following input parameters: effect size f = 0.25, α = 0.05, power = 0.80, number of groups = 2, number of measurements = 3, and non-sphericity correction ε = 1, the analysis indicated that a minimum of 14 participants would be needed to achieve power of at least 0.80. The present study included 27 participants, which exceeds this requirement and indicates adequate statistical power.

## 3. Results

We analyzed the control accuracy using three polar coordinate parameters ΔR (radial position difference), Δθ (angular displacement difference), and Δω (angular velocity difference) obtained based on the presence or absence of the stick in the CES.

### 3.1. Control Accuracy in Terms of ΔR with the Presence of the Stick in CES

**Figure 3:**
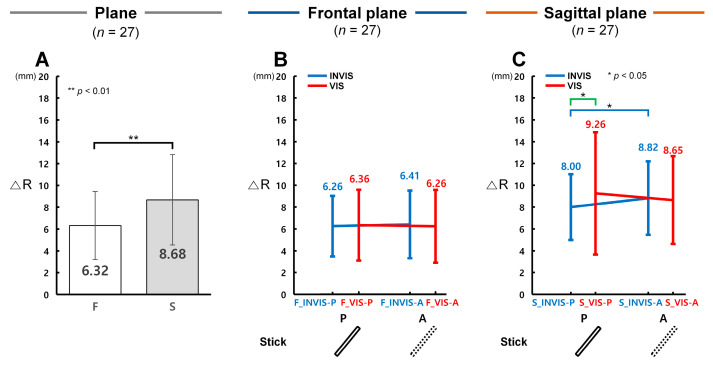
Control accuracy measured by radial position difference (ΔR) under stick conditions in the CES. (**A**) Control accuracy for ΔR in the frontal and sagittal plane. (**B**) Control accuracy for ΔR under the INVIS-P, INVIS-A, VIS-P, and VIS-A sub-conditions in the frontal plane. ∆R is 6.26 ± 2.78, 6.41 ± 3.10, 6.36 ± 3.27, and 6.26 ± 3.35 mm at F_INVIS-P, F_INVIS-A, F_VIS-P, and F_VIS-A, respectively. (**C**) Control accuracy for ΔR under the INVIS-P, INVIS-A, VIS-P, and VIS-A sub-conditions in the sagittal plane. ∆R is 8.00 ± 3.01, 8.82 ± 3.36, 9.26 ± 5.60, and 8.65 ± 4.02 mm at S_INVIS-P, S_INVIS-A, S_VIS-P, and S_VIS-A, respectively. Here, P refers to the presence of the stick and A refers to the absence of the stick.

In terms of distance, control accuracy was significantly higher in the sagittal plane under the INVIS condition when the stick was present (INVIS-P), whereas no significant difference was observed in the frontal plane. Figure 3A presents the analysis results of ΔR values obtained from the CTM, which were used to evaluate distance control accuracy in both the frontal and sagittal planes. Figure 3B and Figure 3C illustrate ΔR results for the INVIS-P, INVIS-A, VIS-P, and VIS-A sub-conditions in the frontal and sagittal planes, respectively. A lower ΔR indicates higher control accuracy in terms of radial distance.

As shown in Figure 3A, the ΔR in the frontal plane (*F*, *M* = 6.32 mm, *SD* = 3.31 mm) was 1.37 times lower than in the sagittal plane (*S*, *M* = 8.68 mm, *SD* = 4.41 mm), and this difference was statistically significant (*F*(1, 159) = 11.399, *p* = 0.001, partial *η*^2^ = 0.067; Item A in Table S1). Among the four conditions, the greatest difference between planes was observed in the VIS-P condition, where the ΔR in the frontal plane was 1.46 times lower than in the sagittal plane. The other conditions showed smaller differences: 1.28 times in INVIS-P, 1.38 times in INVIS-A, 1.38 times in VIS-A). As illustrated in Figure 3B, no statistically significant differences in ΔR were found among the conditions within the frontal plane (F_INVIS-P: *M* = 6.26 mm, *SD* = 2.78 mm; F_INVIS-A: *M* = 6.41 mm, *SD* = 3.10 mm; F_VIS-P: *M* = 6.36 mm, *SD* = 3.27 mm; F_VIS-A: *M* = 6.26 mm, *SD* = 3.35 mm). In contrast, Figure 3C shows that in the sagittal plane, ΔR was lowest in the S_INVIS-P condition (*M* = 8.00 mm, *SD* = 3.01 mm) and highest in the S_VIS-P condition (*M* = 9.26 mm, *SD* = 5.60 mm). The ΔR in S_INVIS-P was 1.10 times lower than in S_INVIS-A (*M* = 8.82 mm, *SD* = 3.36 mm), and this difference was statistically significant (*t*(26) = 2.97, *p* = 0.021, Cohen’s *d* = 0.57; Item B in Table S1), indicating that distance control accuracy was higher when the stick was present in the INVIS condition. Furthermore, ΔR in S_INVIS-P was 1.16 times lower than in S_VIS-P, and this difference was also statistically significant (*t*(26) = 2.99, *p* = 0.019, Cohen’s *d* = 0.58; Item B in Table S1). In the VIS condition, the ΔR in S_VIS-P was 1.07 times higher than in S_VIS-A (*M* = 8.65 mm, *SD* = 4.02 mm), but this difference was not statistically significant.

### 3.2. Control Accuracy in Terms of Δθ with the Presence of the Stick in CES

**Figure 4:**
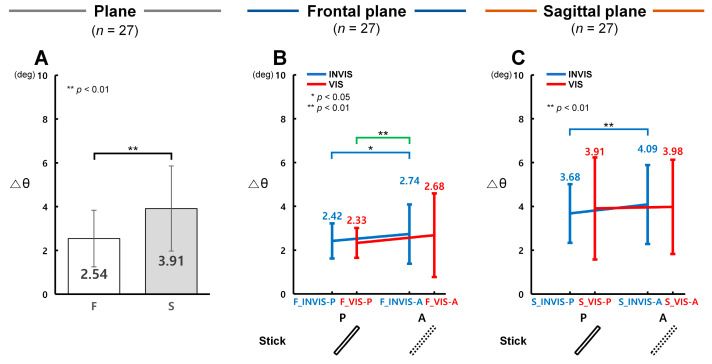
Control accuracy measured by angular displacement difference (Δθ) under stick conditions in the CES. (**A**) Control accuracy for Δθ in the frontal and sagittal plane. (**B**) Control accuracy for Δθ under the INVIS-P, INVIS-A, VIS-P, and VIS-A sub-conditions for the frontal plane. Δθ is 2.42 ± 0.80, 2.74 ± 1.35, 2.33 ± 0.68, and 2.68 ± 1.91° at F_INVIS-P, F_INVIS-A, F_VIS-P, and F_VIS-A, respectively. (**C**) Control accuracy for Δθ under the INVIS-P, INVIS-A, VIS-P, and VIS-A sub-conditions for the sagittal plane. Δθ is 3.68 ± 1.34, 4.09 ± 1.80, 3.91 ± 2.33, and 3.98 ± 2.15° at S_INVIS-P, S_INVIS-A, S_VIS-P, and S_VIS-A, respectively. Here, P refers to the presence of the stick and A refers to the absence of the stick.

In terms of angle (θ), control accuracy was found to be significantly higher under the INVIS condition when the stick was present (INVIS-P) in both the frontal and sagittal planes. Figure 4A presents the analysis results of Δθ obtained from the CTM, evaluating angular control accuracy in both planes. Figure 4B and Figure 4C illustrate Δθ results for the INVIS-P, INVIS-A, VIS-P, and VIS-A sub-conditions in the frontal and sagittal planes, respectively. A lower Δθ indicates higher control accuracy in terms of angle.

As shown in Figure 4A, Δθ in the frontal plane (*F*, *M* = 2.54°, *SD* = 1.29°) was 1.54 times lower than in the sagittal plane (*S*, *M* = 3.91°, *SD* = 1.94°), showing a statistically significant difference (*F*(1, 159) = 14.142, *p* < 0.001, partial η^2^ = 0.082; Item A in Table S2). Among the conditions, the largest difference between planes was observed in VIS-P, where Δθ in the frontal plane was 1.68 times lower than in the sagittal plane. The other conditions showed smaller differences: 1.52 times in INVIS-P, 1.49 times in INVIS-A, and 1.48 times in VIS-A. According to Figure 4B, Δθ in the frontal plane was the lowest in F_VIS-P (*M* = 2.33°, *SD* = 0.68°), and the highest in F_INVIS-A (*M* = 2.74°, *SD* = 1.35°). The Δθ in F_INVIS-P (*M* = 2.42°, *SD* = 0.80°) was 1.13 times lower than in F_INVIS-A, showing a statistically significant difference (*t*(26) = 2.95, *p* = 0.022, Cohen’s *d* = 0.57; Item B in Table S2). Similarly, the Δθ of F_VIS-P, the lowest observed condition, was 1.17 times lower than that of F_INVIS-A, which was also statistically significant (*t*(26) = 3.99, *p* = 0.001, Cohen’s *d* = 0.77; Item B in Table S2). However, the Δθ of F_VIS-P did not differ significantly from that of F_VIS-A (*M* = 2.68°, *SD* = 1.91°). In the sagittal plane results shown in Figure 4C, Δθ was lowest in S_INVIS-P (*M* = 3.68°, *SD* = 1.34°) and highest in S_INVIS-A (*M* = 4.09°, *SD* = 1.80°). The Δθ of S_INVIS-P was 1.11 times lower than that of S_INVIS-A, showing a statistically significant difference (*t*(26) = 3.23, *p* = 0.009, Cohen’s *d* = 0.62; Item B in Table S2). On the other hand, there was no statistically significant difference between S_VIS-P (*M* = 3.91°, *SD* = 2.33°) and S_VIS-A (*M* = 3.98°, *SD* = 2.15°). These results indicate that angular control accuracy was highest in the INVIS-P sub-condition and lowest in the INVIS-A sub-condition, in both the frontal and sagittal planes.

### 3.3. Control Accuracy in Terms of Δω with the Presence of the Stick in CES

**Figure 5:**
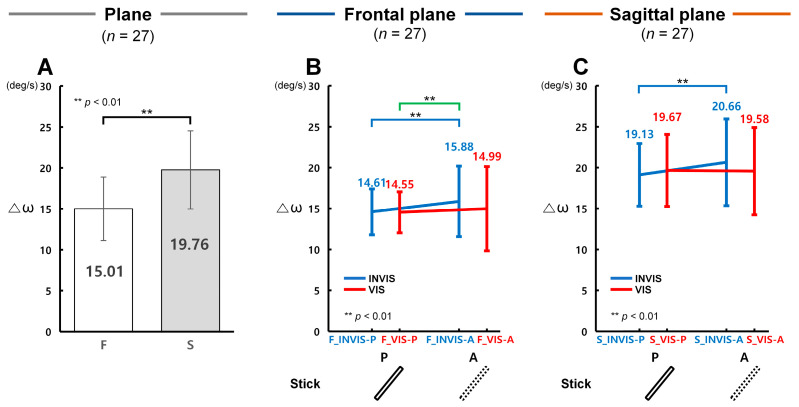
Control accuracy measured by angular velocity difference (Δω) under stick conditions in the CES. (**A**) Control accuracy for Δω in the frontal and sagittal plane. (**B**) Control accuracy for Δω under the INVIS-P, INVIS-A, VIS-P, and VIS-A sub-conditions for the frontal plane. Δω is 14.61 ± 2.80, 15.88 ± 4.31, 14.55 ± 2.51, and 14.99 ± 4.66°/s at F_INVIS-P, F_INVIS-A, F_VIS-P, and F_VIS-A, respectively. (**C**) Control accuracy for Δω under the INVIS-P, INVIS-A, VIS-P, and VIS-A sub-conditions for the sagittal plane. Δω is 19.31 ± 3.80, 20.66 ± 5.27, 19.67 ± 3.89, and 19.58 ± 5.29°/s at S_INVIS-P, S_INVIS-A, S_VIS-P, and S_VIS-A, respectively. Here, P refers to the presence of the stick and A refers to the absence of the stick.

In terms of angular velocity (ω), control accuracy was found to be significantly higher under the INVIS condition when the stick was present (INVIS-P) in both the frontal and sagittal planes. Figure 5A presents the analysis of Δω derived from the CTM, evaluating angular velocity control accuracy in both planes. Figure 5B and Figure 5C illustrate the results of angular velocity control accuracy under the INVIS-P, INVIS-A, VIS-P, and VIS-A sub-conditions in the frontal and sagittal planes, respectively. A lower Δω indicates higher control accuracy in terms of angular velocity.

According to Figure 5A, Δω in the frontal plane (*F*, *M* = 15.01°/s, *SD* = 3.88°/s) was 1.32 times lower than in the sagittal plane (*S*, *M* = 19.76°/s, *SD* = 4.78°/s), showing a statistically significant difference (*F*(1, 159) = 23.997, *p* < 0.001, partial *η*^2^ = 0.131; Item A in Table S3). Among the conditions, the largest difference between planes was observed in VIS-P, where Δω in the frontal plane was 1.35 times lower than in the sagittal plane. The other conditions showed smaller differences: 1.31 times in INVIS-P, 1.30 times in INVIS-A, and 1.31 times in VIS-A. According to Figure 5B, Δω in the frontal plane was lowest in F_VIS-P (*M* = 14.55°/s, *SD* = 2.51°/s), and highest in F_INVIS-A (*M* = 15.88°/s, *SD* = 4.31°/s). Δω in F_INVIS-P (*M* = 14.61°/s, *SD* = 2.80°/s) was 1.09 times lower than in F_INVIS-A, which was statistically significant (*t*(26) = 3.56, *p* = 0.003, Cohen’s *d* = 0.69; Item B in Table S3). Likewise, Δω in F_VIS-P was 1.09 times lower than in F_INVIS-A, also showing a statistically significant difference (*t*(26) = 4.03, *p* = 0.001, Cohen’s *d* = 0.77; Item B in Table S3). In the sagittal plane results shown in Figure 5C, Δω was lowest in S_INVIS-P (*M* = 19.13°/s, *SD* = 3.80°/s) and highest in S_INVIS-A (*M* = 20.66°/s, *SD* = 5.27°/s). Δω in S_INVIS-P was 1.08 times lower than in S_INVIS-A, which was statistically significant (*t*(26) = 3.71, *p* = 0.002, Cohen’s *d* = 0.71; Item B in Table S3). Meanwhile, no statistically significant difference was found between S_VIS-P (*M* = 19.67°/s, *SD* = 3.89°/s) and S_VIS-A (*M* = 19.58°/s, *SD* = 5.29°/s). These results indicate that angular velocity control accuracy was highest in the INVIS-P sub-condition and lowest in the INVIS-A sub-condition in both the frontal and sagittal planes. This pattern was consistent across both angular position (θ) and angular velocity (ω).

## 4. Discussion

This study aimed to quantitatively compare control accuracy in a VR environment based on the presence or absence of a stick and its presentation order in the CES. To this end, we analyzed the tracking performance using ΔR, Δθ, and Δω as outcome variables. We hypothesized that under the INVIS condition, the values of ΔR, Δθ, and Δω would be higher in the absence of the stick (INVIS-A) than in its presence (INVIS-P). As expected, Δθ and Δω in the frontal plane, and ΔR, Δθ, and Δω in the sagittal plane, were significantly increased in the INVIS-A sub-condition (Figure 3C, Figure 4B,C and Figure 5B,C). Similarly, under the VIS condition we expected that the ΔR, Δθ, and Δω values would be lower in the sub-condition where the stick was present (VIS-P) compared to the sub-condition where it was absent (VIS-A). However, under the VIS condition there were no statistically significant differences in either the frontal or sagittal plane (Figure 3B,C, Figure 4B,C and Figure 5B,C). These results suggest that while the presence of the stick improved control accuracy under the INVIS condition, such improvement was only observed when the stick was visible from the beginning. In contrast, under the VIS condition, control accuracy was not influenced by whether the stick appeared later in the trial. The following sections show discussion of the results, with a focus on three main aspects: (1) the effect of stick presence as visual information on control accuracy in the CES, (2) a comparison of control accuracy depending on the presentation order of the stick, and (3) limitations and future research in this study.

### 4.1. Differences in Control Accuracy with the Presence of the Stick in CES

In the present study, under the INVIS condition, the sub-condition in which the stick was present (INVIS-P) showed significantly lower values of ΔR, Δθ, and Δω compared to the sub-condition where the stick was absent (INVIS-A). Specifically, in the frontal plane Δθ and Δω were 1.13 and 1.09 times lower, respectively; in the sagittal plane, ΔR, Δθ, and Δω were 1.10, 1.11, and 1.08 times lower, respectively (Figure 3C, Figure 4B,C and Figure 5B,C). These findings suggest that the provision of the stick as visual information enhanced participants’ control accuracy during the CTM task.

Although the interpretation of the present findings remains hypothetical, the results may primarily be explained by (1) changes in the precision of the body coordinate system, (2) alterations in the state of multisensory integration, and (3) variations in control accuracy relative to the amount of available information. Human visuomotor control accuracy is presumed to be associated with the precision of the body coordinate system. When humans control their bodies, they use a body coordinate-based control mechanism [6,7,8,9,10], body coordinates are known to be constructed by the internal model and proprioceptive sensory information collected through, muscles, tendons, joints, and other components [11,12]. Regarding the generation of body coordinates, the human brain contains grid cells and place cells, which are essential for exploring objects and spaces [13,14,15,16,17,18,19,20]. Grid cells, which create a “reference frame” (including spatial coordinates and direction) for determining object locations and planning movements [16,17,18,19,20,39], are thought to be associated with tracking positional and directional changes in internal models. Place cells, which process sensory inputs and link the positions of the “reference frame” [13,14,15,39] are believed to contribute to the body coordinate system by providing proprioceptive sensory information. The “reference frame” is influenced by various sensory modalities, including visual, tactile, and auditory information [20]. In the context of CTM in VR environment, an internal model is required to track the positional changes between the target, representing the goal location, and the tracer, representing the current location. This process necessitates visual perception of the positions of the target and tracer, along with the assignment of a “reference frame” critical for forming the internal model. The precision of the “reference frame” is likely to differ depending on whether the stick is visible or not. The control of the tracer is achieved through the use of a controller. It is hypothesized that the creation of body coordinates integrates the “reference frame” required for internal model generation with proprioceptive sensory information from the hand and arm holding the controller. In other words, the precision with which body coordinates are generated and adjusted for the circular tracking trajectory is likely a critical determinant of control accuracy.

Multisensory integration of visual and tactile information may also have contributed to the improvement of visuomotor control accuracy. In the INVIS condition, a 20 cm-long white stick was visually attached to the tracer in the VR environment until the third revolution (INVIS-P; Figure 3A), but it disappeared from the tracer starting from the fourth revolution (INVIS-A; Figure 3B). From the participants’ perspective, during the INVIS-P phase, they visually perceived the stick as being attached to the tracer, while simultaneously sensing the controller through tactile feedback. In other words, because visual and tactile information are integrated to control the tracer, the brain likely interprets the controller as being attached to the tracer within the VR environment. The stick may function as an element of the virtual hand illusion (VHI), analogous to the rubber hand illusion (RHI), in which an artificial limb is perceived as part of the body schema, thereby linking the virtual environment with the real world [31,40,41,42]. Another effect of multisensory integration has been reported by Zhou and Popescu (2023) [43], demonstrating that the use of a virtual stick combined with tactile feedback improves perceptual accuracy of virtual forms. This state of multisensory integration may have enhanced the sense of immersion in the VR environment, potentially leading to improved accuracy in controlling the tracer. Multisensory integration may also have contributed to enhancing the precision of the body coordinate system. For example, in the INVIS-P sub-condition, the brain may refine this coordinate system from approximately 5 mm to 1 mm by integrating visual and tactile information. In other words, the higher control accuracy observed when the stick was visible may be attributed to multisensory integration of visual and tactile information, which enhances the precision of the body coordinate system. This may provide a more precise “reference frame” for perceiving the displacement between the target and the tracer.

Furthermore, the stick may have contributed to enhancing control accuracy by supporting the reconstruction of an extended body image from the participant’s hand to the tracer. In the VR environment, participants were required to control the tracer located at the tip of the virtual stick, effectively extending the boundaries of control from the hand to include the length of the stick and the radius of the tracer. A similar phenomenon has been observed in primate studies, where monkeys trained to use a hook over a two-week period were able to retrieve food items that had previously been out of reach. This behavior has been interpreted as the result of body image reconstruction, in which the hook became embodied as an extension of the arm [44,45,46,47,48]. In the present study, the stick may have served a comparable function, acting as a mediating element that facilitated the development of an extended body schema. However, the lower control accuracy observed when the stick was absent in the INVIS-A sub-condition is likely attributable to the loss of visual information necessary for control, the breakdown of multisensory integration, and the resulting decrease in the precision of the internal reference frame.

Finally, this study found that the stick had a substantial influence on the temporal aspects of motor control in the CTM task. A statistically significant effect of stick presence on the spatial distance index ΔR was observed exclusively in the sagittal plane, the temporal indices Δθ and Δω exhibited similar trends and statistically significant differences in both the frontal and sagittal planes (Figure 3B,C, Figure 4B,C and Figure 5B,C). Previous research has reported that, in CTM tasks, Δθ and Δω are more sensitive than ΔR when the target speed is slow [31]. A slower target speed entails less informational change, and under such conditions, participants may perceive little difference when the stick disappears, especially in the INVIS condition, since the tracer continues to closely follow the movement of the controller even without the stick being visible. Nevertheless, the present study revealed statistically significant increases in Δθ and Δω despite this low-speed condition. These results suggest that the stick should be considered an essential visual element for precise control. For instance, in preoperative VR simulation training for high-precision surgeries such as brain surgery, displaying the virtual hand holding the surgical tool could improve the accuracy of performance assessment. Furthermore, in actual surgical settings where simulation outcomes are transferred into real operations, differences in control precision shaped by the presence or absence of visual information during training could directly influence surgical success and even patient survival.

### 4.2. Comparison of Control Accuracy Based on the Order of Stick Visibility in CTM

Control accuracy in CTM in a VR environment appears to be more strongly affected under the INVIS condition. In the INVIS condition, where the stick was initially present (INVIS-P) but later was absent (INVIS-A), the means of ΔR, Δθ, and Δω tended to increase in both the frontal and sagittal planes. All of these increases were statistically significant except for ΔR in the frontal plane (Figure 3C, Figure 4B,C and Figure 5B,C). In contrast, under the VIS condition, where the stick was present later in the trial, no statistically significant decrease was observed in the means of ΔR, Δθ, and Δω when comparing the initial sub-condition without the stick (VIS-A) to the later sub-condition with the stick (VIS-P) (Figure 3B,C, Figure 4B,C and Figure 5B,C). The lower control accuracy observed in the INVIS-A condition may be attributed to the disruption of key control components—such as the reference frame, internal model, and body coordinate system—which had been precisely established when the stick was present. The sudden absence of the stick likely created a lack of reference cues, thereby impairing the precision maintained by the brain’s grid cells and place cells. In contrast, under the VIS condition, no consistent trend or statistically significant difference was observed, and thus it cannot be concluded that control accuracy improved when the stick was present. This may be because the brain had already adapted to performing the circular tracking task without a visual reference such as the stick, forming control-related components in a less precise manner. From the perspective of the brain, even though no visual reference was available, participants likely adapted to the tracking task over time. As a result, the reappearance of the stick may have had only a minimal impact on improving control accuracy. To evaluate how the presentation order of visual information (i.e., the stick) influenced control accuracy in terms of stability, we analyzed differences in standard deviation and conducted Levene’s tests for homogeneity, as shown in Table 3. In the INVIS condition, the increased standard deviations of ΔR, Δθ, and Δω in the frontal and sagittal planes during the INVIS-A, compared to INVIS-P, suggest that the absence of the stick may have led to reduced stability in visuomotor control. The standard deviations of Δθ in both the frontal and sagittal planes, as well as Δω in the sagittal plane, were found to be statistically significant, indicating meaningful differences in movement variability across conditions (Table 3). In contrast, in the VIS condition, the standard deviations of ΔR, Δθ, and Δω in the frontal plane, as well as Δω in the sagittal plane, increased during the VIS-A compared to the VIS-P. This pattern mirrors the trend observed in the INVIS condition, suggesting a decrease in movement stability when visual information was absent. However, in the VIS condition, these differences were not statistically significant (Table 3).

**Figure 6:**
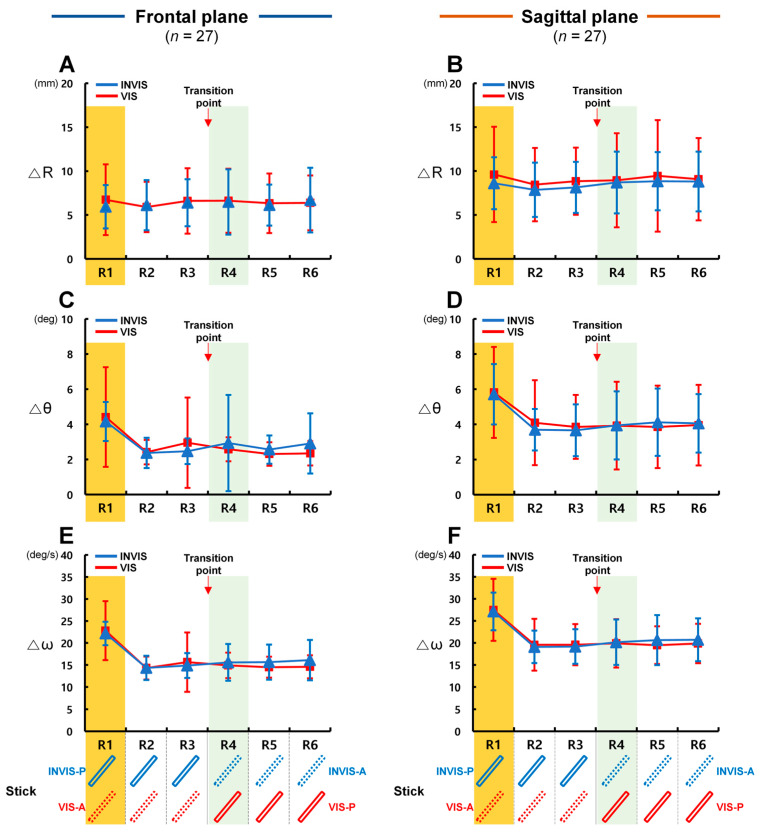
Control accuracy in terms of ΔR, Δθ, Δω by number of revolutions. (**A**) ΔR in the frontal plane, (**B**) ΔR in the sagittal plane, (**C**) Δθ in the frontal plane, (**D**) Δθ in the sagittal plane, (**E**) Δω in the frontal plane, (**F**) Δω in the sagittal plane. R1 ~ R6, 1st revolution ~ 6th revolution. The transition point indicates the point at which the stick’s presence changes (INVIS: presence → absence; VIS: absence → presence).

The effect of the stick’s presentation order on control accuracy appeared to be more pronounced in the sagittal plane than in the frontal plane. By analyzing the results based on the number of revolutions, we were able to better understand the patterns of control accuracy. As illustrated in Figure 2, we designed the experiment to assess how the presence or absence of the stick affected control accuracy during six revolutions of circular tracking under the INVIS and VIS conditions. However, as shown in Figure 6, when ΔR, Δθ, and Δω were analyzed for each revolution under the INVIS and VIS conditions in both the frontal and sagittal planes, an initial higher error rate was observed for Δθ and Δω during the first revolution (R1), unlike for ΔR. Specifically, Δθ and Δω consistently exhibited their highest values in R1 (Figure 6C–E). From the second revolution (R2) to the sixth revolution (R6), their values decreased to an average of 67.0% of R1. Individually, the largest reduction was 52.4% for Δθ in fifth revolution (R5) under the VIS condition in the frontal plane, and the smallest was 76.3% for Δω in R5 under the INVIS condition in the sagittal plane (Table S4). In CTM, the radial distance related to ΔR (radius = 150 mm, Figure 2) could be visually confirmed prior to movement initiation, allowing participants to anticipate and control the position of the tracer. In contrast, angle (Δθ) and angular velocity (Δω) cannot be anticipated in advance unless a predictive movement strategy is used, similar to false starts in track and field events that occur when athletes react before the starting signal. Furthermore, due to neural transmission delays, sensory processing time, and neuromuscular refractory periods, the human visuomotor control system exhibits a processing delay of approximately 200 ms [49,50,51], which likely contributed to the high Δθ and Δω values observed in R1. Because such initial error conditions are no longer present from R2 onward, R1 was excluded from further analysis in order to isolate the effects of stick presence.

In addition, the fourth revolution (R4), which corresponded to the point at which the sub-condition changed and was symmetrical to R1, was also excluded from the analysis. Before excluding R4, we further compared the ANOVA results for the means of R4–6, R4–5, and R5–6 in the INVIS-A and VIS-P sub-conditions, confirming that no changes in trend were observed (Table S5). As a result, under the INVIS condition, values of ΔR, Δθ, and Δω were all significantly lower in INVIS-P than in INVIS-A in the sagittal plane (Figure 3C, Figure 4C and Figure 5C). In the frontal plane, however, significant differences were observed only for Δθ and Δω (Figure 4B and Figure 5B). This suggests that CTM in the sagittal plane, which involves control over depth-related changes, benefited more from the presence of the stick in the INVIS-P sub-condition. Had R1 not been excluded from the analysis, the results may have shown a reversed pattern, with *INVIS-A* appearing to have higher control accuracy than INVIS-P. A comparison of standard deviations across individual revolution indicated reduced variability when the stick was present, with the exception of ΔR and Δθ in the sagittal plane under the VIS condition, even when R1 and R4 were included (Table S4). These findings suggest that displaying the stick provides a more stable control environment during circular tracking.

### 4.3. Further Research and Limitations

This study examined how the presence and order of visual stick presentation influenced control accuracy, as measured by ΔR, Δθ, and Δω in polar coordinates. However, because the experiment was conducted in a fixed order, caution is warranted when interpreting differences in control accuracy according to stick presentation order, and the potential influences of learning and fatigue cannot be fully excluded. Such a sequential procedure may lead to order effects, in which certain conditions are consistently positioned earlier or later, thereby confounding the interpretation of differences between conditions. To enhance the reliability of the findings, several additional examinations were performed.

The potential impact of execution order between planes was first considered. Analyses of ΔR, Δθ, and Δω (Figure 3A, Figure 4A and Figure 5A) revealed significantly higher values in the sagittal plane. Even if learning effects were present, they could not offset these differences, suggesting that control accuracy was higher in the frontal plane than in the sagittal plane. Regarding fatigue, the experimental design included a low task load, short execution time, and sufficient rest intervals, which likely minimized its influence. Previous studies have shown that low loads and short trial durations reduce the likelihood of cumulative fatigue, and that a one-minute micro-break can significantly alleviate fatigue [52,53,54]. In this study, participants used a lightweight controller (153 g) to perform 24-s trials, each consisting of six revolutions, with approximately one minute of rest between trials. Thus, the accumulation of physical fatigue was likely minimized. Nevertheless, unlike studies such as Iqbal et al. (2021) [55], this study did not objectively measure physical fatigue through participant questionnaires or quantify cognitive workload using tools such as the NASA Task Load Index (NASA-TLX), which remains a limitation.

The potential influence of condition order was also examined using ANCOVA and comparisons between sub-conditions. Within each condition, the effect of trial number across the three repetitions was assessed by including it as a covariate in ANCOVA, which revealed no statistically significant effects (ΔR: *F*(1, 159) = 0.824, *p* = 0.365; Δθ: *F*(1, 159) = 0.098, *p* = 0.755; Δω: *F*(1, 159) = 1.835, *p* = 0.177; Table S6). Furthermore, the influence of performing the VIS condition after the INVIS condition was evaluated by comparing INVIS-A and VIS-A, both of which involved the absence of the stick. If significant differences had been observed between these two sub-conditions, they would have indicated learning or fatigue effects. However, analyses of ΔR, Δθ, and Δω showed no statistically significant differences (Figure 3B,C, Figure 4B,C and Figure 5B,C), suggesting that the impact of the fixed sequence was limited. While the fixed order prevents drawing firm conclusions about the absence of stick effects in the VIS condition, the findings indicate that, in the INVIS condition, control accuracy was higher when the stick was present (INVIS-P) than when it was absent (INVIS-A).

Future studies should address these limitations by randomizing the order of condition presentation or applying counterbalancing techniques such as a Latin-square design, ensuring that each condition has an equal probability of appearing earlier or later. Implementing automatic randomization functions within the experimental software would further help reduce experimenter involvement, minimize learning and fatigue effects, and strengthen the validity and generalizability of the findings.

This study was conducted using the HTC Vive HMD and controller. Although several system-related limitations exist, we sought to minimize their impact by carefully considering the experimental design, environmental setup, procedures, and data analysis. First, in the experimental environment, occlusion or reflective surfaces can temporarily reduce the accuracy of posture estimation. The VR device estimates the user’s head and body position and orientation through the HMD and controllers; however, occlusion or reflective surfaces may introduce errors in the estimated values. Such tracking-related instability has been reported to be highly sensitive to the placement of base stations and environmental conditions [56,57]. To prevent this, we securely fixed the base stations and removed any reflective objects or obstructions from the environment. Second, due to the 90 Hz display/processing rate and intermittent reprojection, motion-to-photon latency may occur, which can affect the quantitative estimation of high-speed movements and contribute to cybersickness [58,59]. Reprojection primarily arises during head rotations; therefore, to minimize this issue, we used a custom experimental chair with a belt to stabilize the participant’s upper body. Third, due to the characteristics of the Fresnel optics used in the HMD lenses, the sweet spot is limited and glare may occur, thereby reducing the contrast and readability of visual stimuli [60]. To address this, the target and tracer in the CES were presented within the central visual field, and were displayed as red and yellow spheres against a black background to enhance visibility. Lastly, in VR environments, slight discrepancies in coordinate origins may occur across sessions, which is a structural limitation of the SteamVR tracking system [56,57]. To minimize this coordinate alignment issue, the center position of the CTM was readjusted at the beginning of each trial in accordance with the procedures described by Jo et al. (2020) [10] and Lee et al. (2020) [34].

The provision of a virtual stick in the form of a rod was found to enhance the accuracy and stability of visuomotor control in the brain. This effect appears to result from multisensory integration extended into the VR environment, along with improved precision of the body coordinate system constructed from internal models and proprioceptive information. Future studies will extend this work by introducing a broader range of mediating objects, such as parallel sticks not physically linked to the tracer, as well as geometric forms with lower spatial continuity, including rectangles. Moreover, while the present study focused on control accuracy at a fixed target speed of 0.25 Hz, upcoming experiments will investigate whether similar patterns emerge across varying the target movement speeds, both slower and faster.

Finally, this study was conducted exclusively with participants in their twenties, which limits the generalizability of the findings. Future research should recruit participants across a broader range of age groups to enhance the robustness and applicability of the results. In this regard, Nuanmeesri (2024) [61] demonstrated the value of involving multiple generations by analyzing performance and satisfaction in a study based on Affordable Virtual Learning (AVL) Technology, underscoring the importance of including diverse age groups in similar research contexts.

## 5. Conclusions

This study explained why the presence of a stick enhances control accuracy in visuomotor tasks, drawing on prior findings in neuroscience. In the CES environment, control accuracy and stability were higher when the stick was present than when it was absent. Specifically, distance-based control accuracy in the sagittal plane improved (ΔR 1.10 times lower), and control accuracy for angle and angular velocity improved in the frontal plane (Δθ 1.17 times lower; Δω 1.09 times lower) and in the sagittal plane (Δθ 1.11 times lower; Δω 1.08 times lower). These improvements can be interpreted as increased precision of the body-centered coordinate system achieved by integrating visual information from the stick (rendered as connected to the tracer) with proprioceptive feedback from the handheld controller. Based on these findings, applications in virtual reality that require fine motor control, such as training programs, gaming, and surgical simulation, should render the position of tools like the stick so that users can perceive them or view them as if held in the hand, which may enable more precise control and facilitate goal achievement.

## Figures and Tables

**Table 1:** List of studies utilizing the CES.

Study	Summary	Components	Parameters
Choi et al. (2018) [31]	Comparison of visuomotor control in monocular and binocular vision	Visual field (monocular vs. binocular)	ΔR
Choi et al. (2020) [33]	Analysis of spatial–temporal parameter differences according to the body plane and target speed	Plane of body, velocity of target	ΔR, Δθ, Δω
Jo et al. (2020) [10]	Comparison of visuomotor control between dominant and non-dominant hands in terms of polar kinematic parameters and dimensionless squared jerk (DSJ)	Hand usage (dominant vs. nondominant)	ΔR, Δθ, Δa, dimensionless squared jerk (DSJ)
Lee at al. (2020) [34]	Visuomotor control assessment and strategy investigation based on depth information in the quadrant sections of the sagittal plane	Sagittal plane	ΔR, Δω
Park et al. (2020) [35]	Comparison of circular tracking movement characteristics between the dominant and non-dominant hands through transient response analysis	Transient response analysis and hand usage	Normalized initial peak velocity (IPV2), initial peak time (IPT2), time delay (TD2)
Choi et al. (2021) [32]	Visuomotor control analysis based on visible and invisible circular trajectories at different target speeds	Orbit	Δθ, Δω
Kim et al. (2023) [36]	Comparison of 3D tracking performance based on the rotation axis of the circular trajectory	Sagittal plane (0, 45, 90 deg)	ΔR
Ours	Comparison of control accuracy based on the presence of a stick and its presentation order	Stick	ΔR, Δθ, Δω

**Table 2:** Measurement parameters.

No.	Parameter	Image	Formula
1	Radial position difference	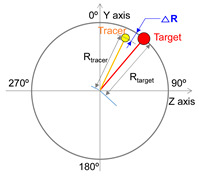	$△R\left[mm\right]=\frac{\sum_{t=1}^{n}abs(R_{tracer}\left(t\right)-R_{target}\left(t\right))}{n}$
2	Angular displacement difference	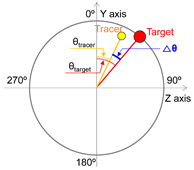	$△\theta \left[deg\right]=\frac{\sum_{t=1}^{n}abs(\theta_{tracer}\left(t\right)-\theta_{target}\left(t\right))}{n}$
3	Angular velocity difference	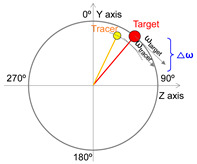	$△\omega \left[\frac{deg}{s}\right]=\frac{\sum_{t=1}^{n}abs(\omega_{tracer}\left(t\right)-\omega_{target}\left(t\right))}{n}$

**Table 3:** Comparison of standard deviation (*SD*) according to the presence (P) or absence (A) of the stick in each condition (INVIS and VIS), with statistical significance assessed using Levene’s test. (* *p* < 0.05).

State	Plane	Variable	State of the Stick		ΔSD (A-P)	Levene’s *p*-Value
			P	A		
INVIS	Frontal	ΔR	2.78	3.10	+0.32	0.875
		Δθ	0.80	1.35	+0.55	0.031 *
		Δω	2.80	4.31	+1.51	0.063
	Sagittal	ΔR	3.01	3.36	+0.35	0.304
		Δθ	1.34	1.80	+0.46	0.017 *
		Δω	3.82	5.30	+1.48	0.021 *
VIS	Frontal	ΔR	3.27	3.35	+0.08	0.807
		Δθ	0.68	1.91	+1.23	0.059
		Δω	2.51	5.15	+2.64	0.139
	Sagittal	ΔR	5.60	4.02	−1.58	0.158
		Δθ	2.33	2.15	−0.18	0.841
		Δω	4.40	5.33	+0.93	0.106

## Data Availability

All raw and processed data are available via the OSF repository at the following DOI: 10.17605/OSF.IO/QPNWZ.

## Supplementary Materials

The following are available online at https://www.mdpi.com/article/10.3390/s25195998/s1, Table S1: Summary of statistical analysis results for ΔR, Table S2: Summary of statistical analysis results for Δθ, Table S3: Summary of statistical analysis results for Δω, Table S4: Analysis of mean and standard deviation by number of revolutions, Table S5: Results of ANOVA comparing mean values of ΔR, Δθ, and Δω, Table S6: Repeated-measures ANCOVA of ΔR, Δθ, and Δω with trial and revolution as covariates.
