# Supplementary material for: Visuomotor Control Accuracy of Circular Tracking Movement According to Visual Information in Virtual Space
Source: Sensors (Basel). 2025 Sep 29;25(19):5998. doi: 10.3390/s25195998 (PMC12526675; doi:10.3390/s25195998)
Supplement: Supplementary file 1 [file sensors-25-05998-s001.zip › Table S1. Summary of statistical analysis results for ΔR.pdf]

Table S1. Summary of statistical analysis results for ΔR

Item	Variable	Test	Statistic	Confidence
A	ΔR between the plane and state	Two-way repeated measures ANCOVA	plane:<br>Mauchly's Test$\chi^2(0) = 0$,<br>p = Nothing, $\varepsilon = 1$;<br>$F(1, 159) = 11.399$;<br><br>state:<br>Mauchly's Test$\chi^2(5) = 35.316$,<br>$p = 0.000$, $\varepsilon = 0.863$;<br>$F(2.668, 424.143) = 1.501$;<br><br>plane×state interaction:<br>Mauchly's Test$\chi^2(5) = 35.898$,<br>$p = 0.000$, $\varepsilon = 0.858$;<br>$F(2.654, 421.966) = 0.175$;	plane: $p = 0.001$, partial $\eta^2 = 0.067$<br><br>state: $p = 0.218$, partial $\eta^2 = 0.009$<br><br>plane×state interaction:<br>$p = 0.893$, partial $\eta^2 = 0.001$
B	ΔR under the conditions of state at plane	Bonferroni-corrected pairwise comparisons	INVIS-P and INVIS-A at frontal plane<br>$t(26) = 0.77$;<br><br>INVIS-P and VIS-P at frontal plane<br>$t(26) = 0.43$;<br><br>INVIS-P and VIS-A at frontal plane<br>$t(26) = 0.01$;<br><br>INVIS-A and VIS-P at frontal plane<br>$t(26) = 0.26$;<br><br>INVIS-A and VIS-A at frontal plane<br>$t(26) = 0.68$;<br><br>VIS-P and VIS-A at frontal plane<br>$t(26) = 0.42$;<br><br>INVIS-P and INVIS-A at sagittal plane<br>$t(26) = 2.97$;<br><br>INVIS-P and VIS-P at sagittal plane<br>$t(26) = 2.99$;<br><br>INVIS-P and VIS-A at sagittal plane<br>$t(26) = 2.16$;<br><br>INVIS-A and VIS-P at sagittal plane<br>$t(26) = 1.22$;<br><br>INVIS-A and VIS-A at sagittal plane<br>$t(26) = 0.63$;<br><br>VIS-P and VIS-A at sagittal plane<br>$t(26) = 1.86$;	INVIS-P and INVIS-A at frontal plane<br>$p = 1.000$, Cohen's $d = 0.15$;<br><br>INVIS-P and VIS-P at frontal plane<br>$p = 1.000$, Cohen's $d = 0.08$;<br><br>INVIS-P and VIS-A at frontal plane<br>$p = 1.000$, Cohen's $d = 0.00$;<br><br>INVIS-A and VIS-P at frontal plane<br>$p = 1.000$, Cohen's $d = 0.05$;<br><br>INVIS-A and VIS-A at frontal plane<br>$p = 1.000$, Cohen's $d = 0.13$;<br><br>VIS-P and VIS-A at frontal plane<br>$p = 1.000$, Cohen's $d = 0.08$;<br><br>INVIS-P and INVIS-A at sagittal plane<br>$p = 0.021$, Cohen's $d = 0.57$;<br><br>INVIS-P and VIS-P at sagittal plane<br>$p = 0.019$, Cohen's $d = 0.58$;<br><br>INVIS-P and VIS-A at sagittal plane<br>$p = 0.195$, Cohen's $d = 0.42$;<br><br>INVIS-A and VIS-P at sagittal plane<br>$p = 1.000$, Cohen's $d = 0.23$;<br><br>INVIS-A and VIS-A at sagittal plane<br>$p = 1.000$, Cohen's $d = 0.12$;<br><br>VIS-P and VIS-A at sagittal plane<br>$p = 0.388$, Cohen's $d = 0.36$;
