# Supplementary material for: Visuomotor Control Accuracy of Circular Tracking Movement According to Visual Information in Virtual Space
Source: Sensors (Basel). 2025 Sep 29;25(19):5998. doi: 10.3390/s25195998 (PMC12526675; doi:10.3390/s25195998)
Supplement: Supplementary file 1 [file sensors-25-05998-s001.zip › Table S2. Summary of statistical analysis results for Δθ.pdf]

Table S2. Summary of statistical analysis results for Δθ

Item	Variable	Test	Statistic	Confidence
A	Δθ between the plane and state	Two-way repeated measures ANCOVA	plane:<br>Mauchly's Test $\chi^2(0) = 0$,<br>p = Nothing, $\varepsilon = 1$;<br>$F(1, 159) = 14.142$;<br><br>state:<br>Mauchly's Test $\chi^2(5) = 46.849$,<br>$p = 0.000$, $\varepsilon = 0.850$;<br>$F(2.629, 417.965) = 0.160$;<br><br>plane×state interaction:<br>Mauchly's Test $\chi^2(5) = 30.369$,<br>$p = 0.000$, $\varepsilon = 0.881$;<br>$F(2.725, 433.260) = 1.371$;	plane: $p = 0$, partial $\eta^2 = 0.082$<br><br>state: $p = 0.903$, partial $\eta^2 = 0.001$<br><br>plane×state interaction:<br>$p = 0.253$, partial $\eta^2 = 0.009$
B	Δθ under the conditions of state at plane	Bonferroni-corrected pairwise comparisons	INVIS-P and INVIS-A at frontal plane<br>$t(26) = 2.95$;<br><br>INVIS-P and VIS-P at frontal plane<br>$t(26) = 1.28$;<br><br>INVIS-P and VIS-A at frontal plane<br>$t(26) = 1.82$;<br><br>INVIS-A and VIS-P at frontal plane<br>$t(26) = 3.99$;<br><br>INVIS-A and VIS-A at frontal plane<br>$t(26) = 0.31$;<br><br>VIS-P and VIS-A at frontal plane<br>$t(26) = 2.39$;<br><br>INVIS-P and INVIS-A at sagittal plane<br>$t(26) = 3.23$;<br><br>INVIS-P and VIS-P at sagittal plane<br>$t(26) = 1.50$;<br><br>INVIS-P and VIS-A at sagittal plane<br>$t(26) = 2.00$;<br><br>INVIS-A and VIS-P at sagittal plane<br>$t(26) = 1.01$;<br><br>INVIS-A and VIS-A at sagittal plane<br>$t(26) = 0.67$;<br><br>VIS-P and VIS-A at sagittal plane<br>$t(26) = 0.54$;	INVIS-P and INVIS-A at frontal plane<br>$p = 0.022$, Cohen's $d = 0.57$;<br><br>INVIS-P and VIS-P at frontal plane<br>$p = 1.000$, Cohen's $d = 0.25$;<br><br>INVIS-P and VIS-A at frontal plane<br>$p = 0.419$, Cohen's $d = 0.35$;<br><br>INVIS-A and VIS-P at frontal plane<br>$p = 0.001$, Cohen's $d = 0.77$;<br><br>INVIS-A and VIS-A at frontal plane<br>$p = 1.000$, Cohen's $d = 0.06$;<br><br>VIS-P and VIS-A at frontal plane<br>$p = 0.109$, Cohen's $d = 0.46$;<br><br>INVIS-P and INVIS-A at sagittal plane<br>$p = 0.009$, Cohen's $d = 0.62$;<br><br>INVIS-P and VIS-P at sagittal plane<br>$p = 0.815$, Cohen's $d = 0.29$;<br><br>INVIS-P and VIS-A at sagittal plane<br>$p = 0.285$, Cohen's $d = 0.38$;<br><br>INVIS-A and VIS-P at sagittal plane<br>$p = 1.000$, Cohen's $d = 0.19$;<br><br>INVIS-A and VIS-A at sagittal plane<br>$p = 1.000$, Cohen's $d = 0.13$;<br><br>VIS-P and VIS-A at sagittal plane<br>$p = 1.000$, Cohen's $d = 0.10$;
