# Supplementary material for: Visuomotor Control Accuracy of Circular Tracking Movement According to Visual Information in Virtual Space
Source: Sensors (Basel). 2025 Sep 29;25(19):5998. doi: 10.3390/s25195998 (PMC12526675; doi:10.3390/s25195998)
Supplement: Supplementary file 1 [file sensors-25-05998-s001.zip › Table S3. Summary of statistical analysis results for Δω.pdf]

Table S3. Summary of statistical analysis results for Δω

Item	Variable	Test	Statistic	Confidence
A	Δω between the plane and state	Two-way repeated measures ANCOVA	plane:<br>Mauchly's Test$\chi^2(0) = 0$,<br>p = Nothing, $\varepsilon = 1$;<br>$F(1,159) = 23.997$;<br><br>state:<br>Mauchly's Test$\chi^2(5) =$ 54.007,<br>$p = 0.000$, $\varepsilon = 0.801$;<br>$F(2.472,393.058) = 1.011$;<br><br>plane×state interaction:<br>Mauchly's Test$\chi^2(5) =$ 34.292,<br>$p = 0.000$, $\varepsilon = 0.877$;<br>$F(2.712,431.152) = 0.208$;	plane: $p = 0$, partial $\eta^2 = 0.131$<br><br>state: $p = 0.377$, partial $\eta^2 = 0.006$<br><br>plane×state interaction:<br>$p = 0.873$, partial $\eta^2 = 0.001$
B	Δω under the conditions of state at plane	Bonferroni-corrected pairwise comparisons	INVIS-P and INVIS-A at frontal plane<br>$t(26) = 3.56$;<br><br>INVIS-P and VIS-P at frontal plane<br>$t(26) = 0.24$;<br><br>INVIS-P and VIS-A at frontal plane<br>$t(26) = 1.01$;<br><br>INVIS-A and VIS-P at frontal plane<br>$t(26) = 4.03$;<br><br>INVIS-A and VIS-A at frontal plane<br>$t(26) = 1.83$;<br><br>VIS-P and VIS-A at frontal plane<br>$t(26) = 1.16$;<br><br>INVIS-P and INVIS-A at sagittal plane<br>$t(26) = 3.71$;<br><br>INVIS-P and VIS-P at sagittal plane<br>$t(26) = 1.40$;<br><br>INVIS-P and VIS-A at sagittal plane<br>$t(26) = 1.03$;<br><br>INVIS-A and VIS-P at sagittal plane<br>$t(26) = 2.24$;<br><br>INVIS-A and VIS-A at sagittal plane<br>$t(26) = 2.08$;<br><br>VIS-P and VIS-A at sagittal plane<br>$t(26) = 0.24$;	INVIS-P and INVIS-A at frontal plane<br>$p = 0.003$, Cohen's $d = 0.69$;<br><br>INVIS-P and VIS-P at frontal plane<br>$p = 1.000$, Cohen's $d = 0.05$;<br><br>INVIS-P and VIS-A at frontal plane<br>$p = 1.000$, Cohen's $d = 0.20$;<br><br>INVIS-A and VIS-P at frontal plane<br>$p = 0.001$, Cohen's $d = 0.77$;<br><br>INVIS-A and VIS-A at frontal plane<br>$p = 0.414$, Cohen's $d = 0.35$;<br><br>VIS-P and VIS-A at frontal plane<br>$p = 1.000$, Cohen's $d = 0.22$;<br><br>INVIS-P and INVIS-A at sagittal plane<br>$p = 0.002$, Cohen's $d = 0.71$;<br><br>INVIS-P and VIS-P at sagittal plane<br>$p = 0.976$, Cohen's $d = 0.27$;<br><br>INVIS-P and VIS-A at sagittal plane<br>$p = 1.000$, Cohen's $d = 0.20$;<br><br>INVIS-A and VIS-P at sagittal plane<br>$p = 0.161$, Cohen's $d = 0.43$;<br><br>INVIS-A and VIS-A at sagittal plane<br>$p = 0.233$, Cohen's $d = 0.40$;<br><br>VIS-P and VIS-A at sagittal plane<br>$p = 1.000$, Cohen's $d = 0.05$;
