# Supplementary material for: Visuomotor Control Accuracy of Circular Tracking Movement According to Visual Information in Virtual Space
Source: Sensors (Basel). 2025 Sep 29;25(19):5998. doi: 10.3390/s25195998 (PMC12526675; doi:10.3390/s25195998)
Supplement: Supplementary file 1 [file sensors-25-05998-s001.zip › Table S4. Analysis of mean and standard deviation by number of revolutions.pdf]

Table S4. Analysis of mean and standard deviation by number of rotations

			Frontal plane						Sagittal plane					
			R1	R2	R3	R4	R5	R6	R1	R2	R3	R4	R5	R6
ΔR (mm)	Mean	INVIS	P			A			P			A		
			5.94 (-)	6.12 (102.9%)	6.40 (107.7%)	6.48 (109.1%)	6.13 (103.1%)	6.69 (112.6%)	8.62 (-)	7.86 (91.3%)	8.14 (94.4%)	8.69 (100.9%)	8.84 (102.6%)	8.80 (102.2%)
		VIS	A			P			A			P		
			6.74 (-)	5.91 (87.7%)	6.61 (98.1%)	6.62 (98.3%)	6.34 (94.1%)	6.38 (94.7%)	9.62 (-)	8.45 (87.9%)	8.85 (92.0%)	8.95 (93.1%)	9.45 (98.3%)	9.08 (94.4%)
	SD	INVIS	P			A			P			A		
			2.47	2.85	2.68	3.71	2.33	3.68	2.96	3.09	2.91	3.52	3.31	3.39
				M: 2.67			M: 3.24			M: 2.99			M: 3.41	
		VIS	A			P			A			P		
			4.03	2.87	3.73	3.65	3.39	3.12	5.42	4.17	3.82	5.36	6.35	4.68
				M: 3.54			M: 3.38			M: 4.47			M: 5.46	
$\Delta\theta$ (deg)	Mean	INVIS	P			A			P			A		
			4.16 (-)	2.37 (57.0%)	2.47 (59.4%)	2.93 (70.5%)	2.56 (61.6%)	2.91 (70.0%)	5.71 (-)	3.69 (64.7%)	3.66 (64.1%)	3.94 (69.0%)	4.12 (72.1%)	4.05 (71.0%)
		VIS	A			P			A			P		
			4.41 (-)	2.42 (54.7%)	2.95 (66.9%)	2.58 (58.4%)	2.31 (52.4%)	2.35 (53.2%)	5.82 (-)	4.10 (70.4%)	3.86 (66.3%)	3.93 (67.5%)	3.86 (66.3%)	3.96 (68.0%)
	SD	INVIS	P			A			P			A		
			1.11	0.86	0.73	2.74	0.81	1.71	1.72	1.18	1.48	1.94	1.91	1.66
				M: 0.90			M: 1.75			M: 1.46			M: 1.84	
		VIS	A			P			A			P		
			2.83	0.70	2.57	0.69	0.67	0.69	2.59	2.42	1.82	2.50	2.35	2.29
				M: 2.03			M: 0.68			M: 2.27			M: 2.38†	
$\Delta\omega$ (deg/s)	Mean	INVIS	P			A			P			A		
			22.15 (-)	14.34 (64.7%)	14.87 (67.1%)	15.58 (70.4%)	15.66 (70.7%)	16.10 (72.7%)	27.14 (-)	19.09 (70.4%)	19.16 (70.6%)	20.15 (74.2%)	20.62 (76.0%)	20.70 (76.3%)
		VIS	A			P			A			P		
			22.79 (-)	14.31 (62.8%)	15.66 (68.7%)	14.91 (65.4%)	14.50 (63.6%)	14.60 (64.1%)	27.49 (-)	19.57 (71.2%)	19.59 (71.2%)	19.89 (72.3%)	19.48 (70.9%)	19.86 (72.2%)
	SD	INVIS	P			A			P			A		
			2.66	2.74	2.82	4.18	3.98	4.57	4.28	3.69	3.92	5.16	5.67	4.86
				M: 2.74			M: 4.24			M: 3.96			M: 5.23	
		VIS	A			P			A			P		
			6.69	2.60	6.71	2.88	2.38	2.61	7.05	5.90	4.67	5.50	4.29	4.48
				M: 5.33			M: 2.62			M: 5.87			M: 4.76	
