# Supplementary material for: Visuomotor Control Accuracy of Circular Tracking Movement According to Visual Information in Virtual Space
Source: Sensors (Basel). 2025 Sep 29;25(19):5998. doi: 10.3390/s25195998 (PMC12526675; doi:10.3390/s25195998)
Supplement: Supplementary file 1 [file sensors-25-05998-s001.zip › Table S5. Results of ANOVA comparing mean values of ΔR, Δθ, and Δω.pdf]

Table S5. Results of ANOVA comparing mean values of ΔR, Δθ, and Δω across sub-condition revolutions (R4–6, R4–5, R5–6).

State (Sub-condition)	Plane	Variable	*Mean ± SD*			*df*	*F*	*p*
			**R4-6**	**R4-5**	**R5-6**			
INVIS-A	Frontal	ΔR	6.43 ± 2.11	6.31 ± 1.92	6.41 ± 2.26	2, 78	0.029	.972
		Δθ	2.8 ± 0.93	2.75 ± 0.93	2.74 ± 0.83	2, 78	0.041	.960
		Δω	15.78 ± 2.22	15.62 ± 2.11	15.88 ± 2.71	2, 78	0.082	.921
	Sagittal	ΔR	8.78 ± 2.17	8.77 ± 2.18	8.82 ± 2.24	2, 78	0.005	.995
		Δθ	4.04 ± 1.17	4.03 ± 1.25	4.09 ± 1.11	2, 78	0.019	.982
		Δω	20.49 ± 2.98	20.39 ± 3.27	20.66 ± 3.1	2, 78	0.055	.947
VIS-P	Frontal	ΔR	6.45 ± 2.43	6.48 ± 2.5	6.36 ± 2.43	2, 78	0.017	.983
		Δθ	2.41 ± 0.48	2.45 ± 0.5	2.33 ± 0.49	2, 78	0.395	.675
		Δω	14.67 ± 1.83	14.7 ± 1.84	14.55 ± 1.83	2, 78	0.052	.949
	Sagittal	ΔR	9.16 ± 3.41	9.2 ± 3.63	9.26 ± 3.64	2, 78	0.006	.994
		Δθ	3.91 ± 1.43	3.89 ± 1.5	3.91 ± 1.41	2, 78	0.002	.998
		Δω	19.74 ± 3.15	19.69 ± 3.46	19.67 ± 2.84	2, 78	0.004	.996
