# Supplementary material for: Visuomotor Control Accuracy of Circular Tracking Movement According to Visual Information in Virtual Space
Source: Sensors (Basel). 2025 Sep 29;25(19):5998. doi: 10.3390/s25195998 (PMC12526675; doi:10.3390/s25195998)
Supplement: Supplementary file 1 [file sensors-25-05998-s001.zip › Table S6. Repeated-measures ANCOVA of ΔR, Δθ, and Δω with trial and revolution as covariates.pdf]

Table S6. Repeated-measures ANCOVA of ΔR, Δθ, and Δω with trial and revolution as covariates.

**Variable**	**Domain**	**Effect**	***df***	***F***	***p***	**partial η^2**
ΔR	Within-subjects	State × Trial	2.668, 424.143	0.506	.657	0.003
		State × Revolution	2.668, 424.143	1.093	.348	0.007
	Between-subjects	Trial	1, 159	0.824	.365	0.005
		Revolution	1, 159	0.292	.590	0.002
Δθ	Within-subjects	State × Trial	2.629, 417.965	0.441	.698	0.003
		State × Revolution	2.629, 417.965	0.166	.899	0.001
	Between-subjects	Trial	1, 159	0.098	.755	0.001
		Revolution	1, 159	0.387	.535	0.002
Δω	Within-subjects	State × Trial	2.472, 393.058	0.504	.644	0.003
		State × Revolution	2.472, 393.058	0.254	.821	0.002
	Between-subjects	Trial	1, 159	1.835	.177	0.011
		Revolution	1, 159	0.889	.347	0.006

Note. Within-subjects effects are reported with Huynh–Feldt–adjusted degrees of freedom; between-subjects effects use unadjusted df. Sphericity diagnostics
