# Supplementary figures and images for: Visuomotor Control Accuracy of Circular Tracking Movement According to Visual Information in Virtual Space
Source: Sensors (Basel). 2025 Sep 29;25(19):5998. doi: 10.3390/s25195998 (PMC12526675; doi:10.3390/s25195998)

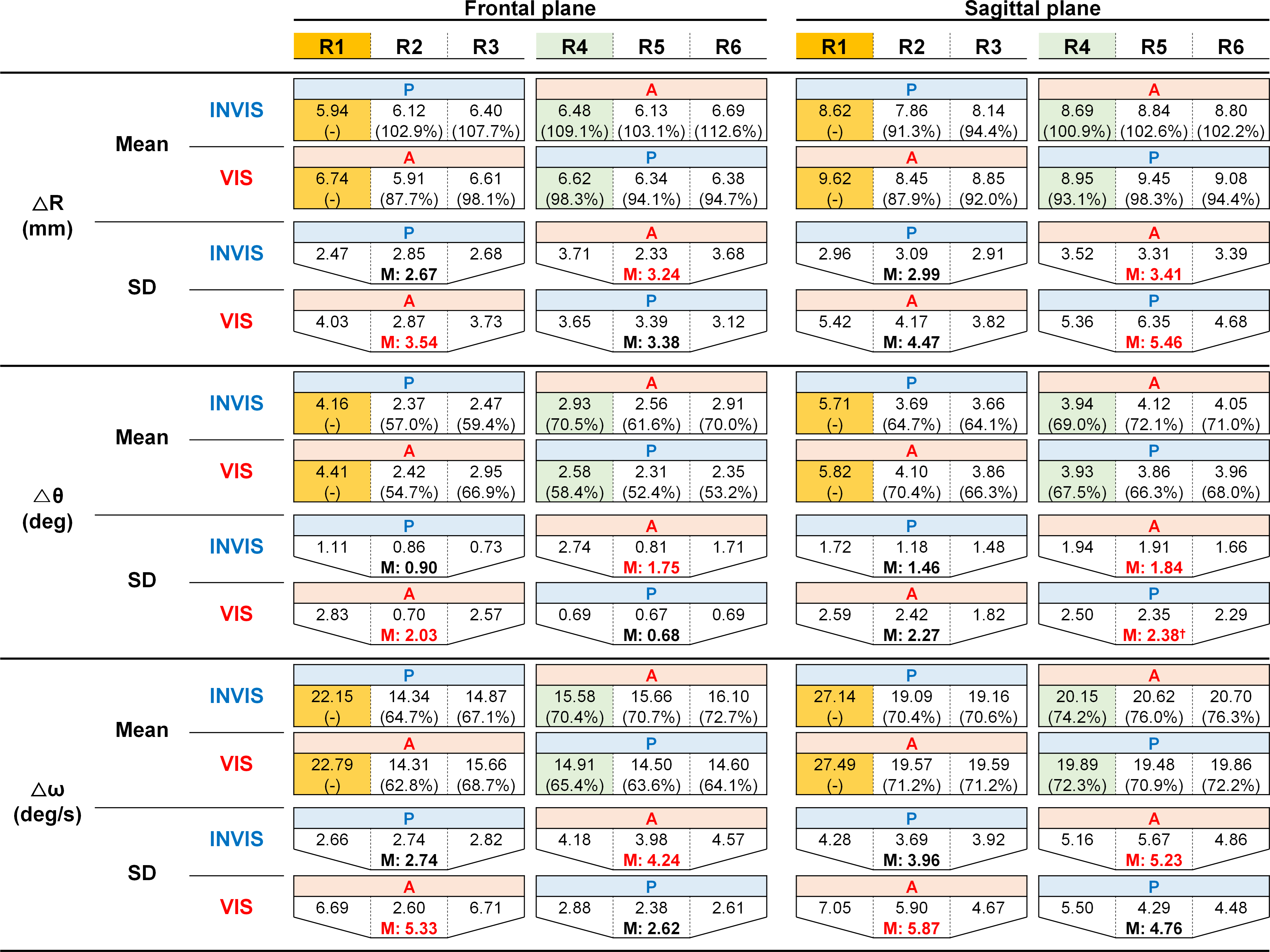

Supplement: Supplementary file 1 [file sensors-25-05998-s001.zip › Table S4.tif]
